# When Cardiac Magnetic Resonance Changed the Diagnosis of a Young Patient With Spontaneous Coronary Artery Dissection

**DOI:** 10.7759/cureus.72075

**Published:** 2024-10-21

**Authors:** Mahmoud A. Ismaiel, Mohamed Halawa, Mohamed Salah Shehata, Ataa Khaleel Taha

**Affiliations:** 1 Cardiology, Kobry El Kobba Medical Complex, Cairo, EGY; 2 Cardiovascular, Faculty of Medicine, Mansoura University, Mansoura, EGY; 3 Cardiology, Cairo University Kasr Alainy Medical College, Cairo, EGY; 4 Cardiovascular, University of Baghdad, Baghdad, IRQ

**Keywords:** acs, cardiac magnetic resonance (cmr), ctca, echo, spontaneous coronary artery dissection

## Abstract

Spontaneous coronary artery dissection (SCAD) is a significant cause of acute coronary syndrome, myocardial infarction, arrhythmia, and sudden death, particularly in young women and individuals with few conventional atherosclerotic risk factors, necessitating a high degree of suspicion. The most common risk factors for SCAD include atherosclerosis, females in the peripartum period, autoimmune inflammatory diseases, and connective tissue diseases. We present an unusual case of a young man who was initially suspected of having myocarditis, but cardiac magnetic resonance (CMR) revealed an ischemic pattern in late gadolinium enhancement. A coronary angiography (CA) led to the diagnosis of SCAD. We report a case of a 28-year-old male who presented with typical chest pain on and off for the past three months. Since the patient had no medical or cardiac history, the previous computed tomography CA (CTCA) revealed normal coronaries, raising the suspicion of myocarditis. Consequently, we performed a CMR, which revealed subendocardial delayed enhancement in the apical anterior and mid-anteroseptal segments, thereby ruling out myocarditis. The decision was made here to perform a coronary angiogram, which led to the diagnosis of SCAD.

## Introduction

Spontaneous coronary artery dissection (SCAD) primarily affects the epicardial vessels and is not caused by atherosclerotic changes, trauma, or iatrogenic events during coronary intervention. SCAD can cause direct myocardial injury through coronary artery obstruction, which results from false lumen obstruction or intramural hematoma formation [1,2]. The availability of multi-image modalities has made it easier to diagnose, understand the epidemiology, and classify SCAD, leading to a higher detection rate than in the past [1-3].

Coronary angiography is the main diagnostic tool of SCAD, and the availability of intracoronary imaging, such as intravascular ultrasound (IVUS) or optical coherence tomography (OCT), makes the diagnosis much easier. cardiac magnetic resonance (CMR) and computed tomography coronary angiography (CTCA) can also play a valuable role in the diagnosis [4,5]. The clinical presentation of the patients is the main determinant of SCAD management: stable patients with preserved coronary blood flow require medical treatment and follow-up, whereas unstable patients, who typically present with manifestations of acute coronary syndrome and significant coronary obstruction, undergo revascularization [4,5].

We present an uncommon case of SCAD in a young male patient, whose diagnosis necessitated multiple imaging modalities. Clinical suspicion and assessment are the most important in the diagnosis, as the investigations are sometimes mistaken.

## Case presentation

We reported that a 28-year-old male patient presented to our hospital on day one complaining of typical chest pain that started three months ago. He does not have any medical or cardiac conditions, and there is no history of cardiac disease in his family. The patient was hemodynamically stable, with a blood pressure of 110/70 mmHg, a heart rate of 55/min regular, and afebrile with room air saturations maintained at 98%. The clinical examination revealed no significant findings.

**Figure 1:**
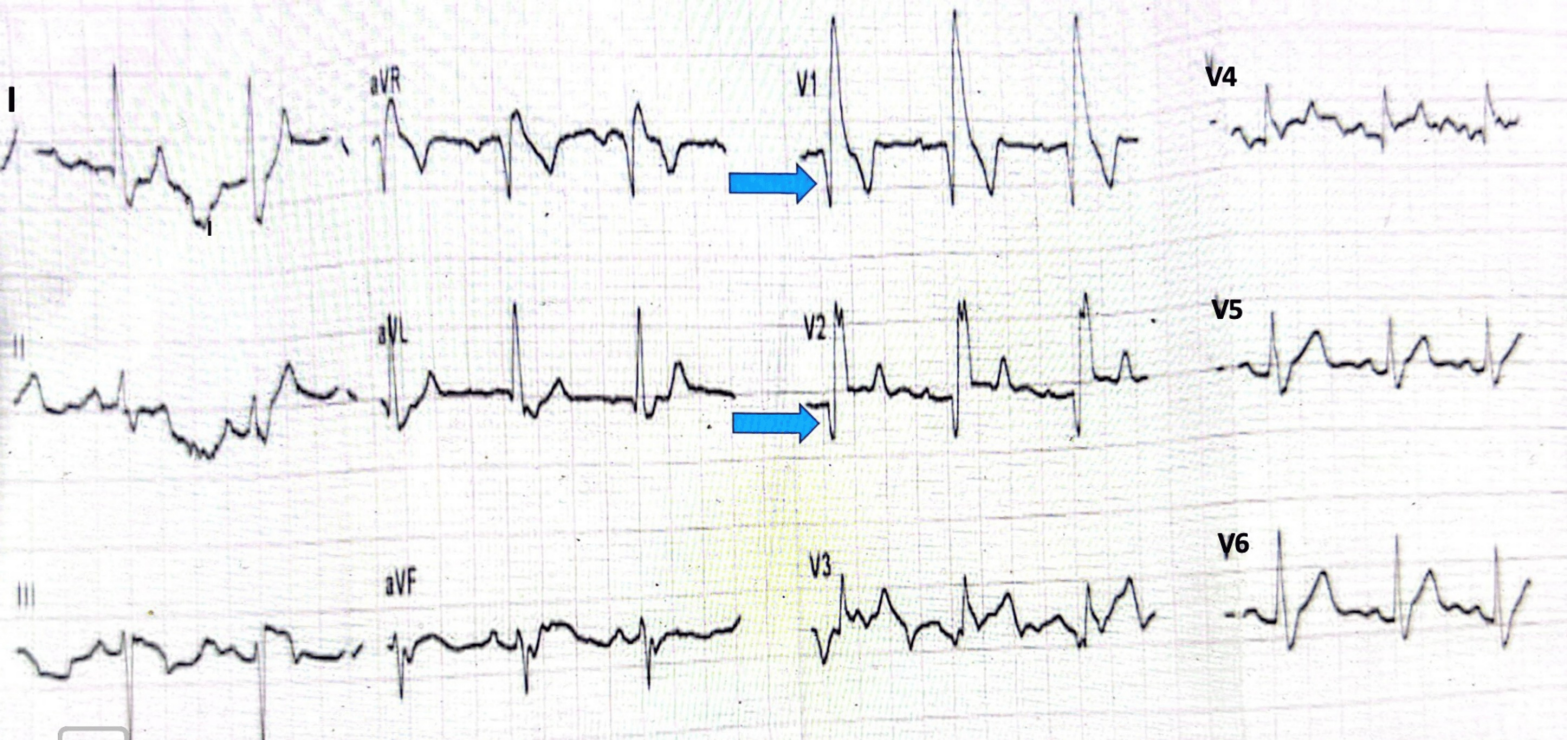
12-Lead resting ECG showing IVCD and a Q wave in lead V1-2.

An electrocardiogram (ECG) was done and showed interventricular conduction delay (IVCD) with a Q wave in lead V1-2 (Figure 1). He was admitted to the coronary care unit (CCU) for further observation. Upon admission, the echocardiography showed a mildly dilated left ventricular internal dimension (LVID, 62 mm) and resting wall motion abnormalities in the apical anterior and anteroseptal hypokinesia, with a mildly reduced ejection fraction of 45%.

**Figure 2:**
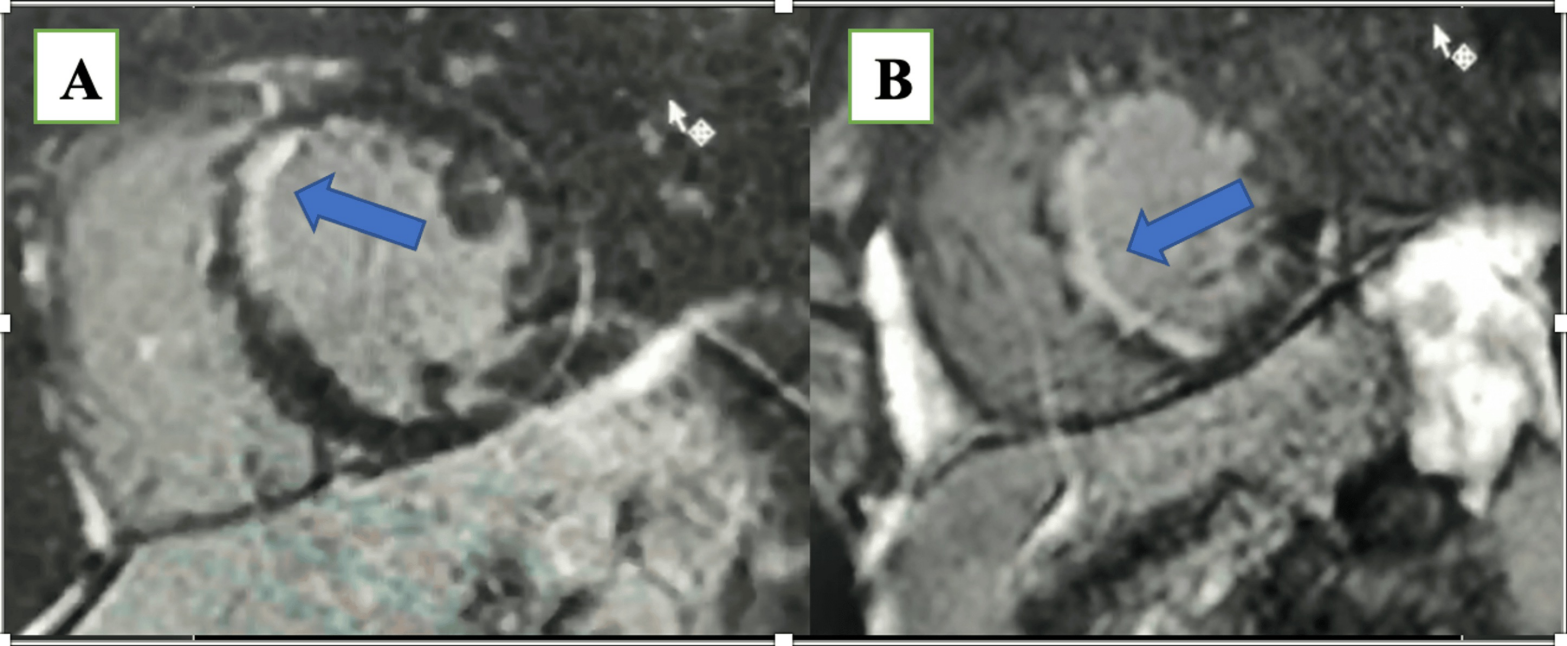
Cardiac magnetic resonance, short-axis view, and late gadolinium enhancement phase, showing subendocardial hyperenhancement in a mid-anteroseptal segment (A) and septal wall of the apical level (B).

**Figure 3:**
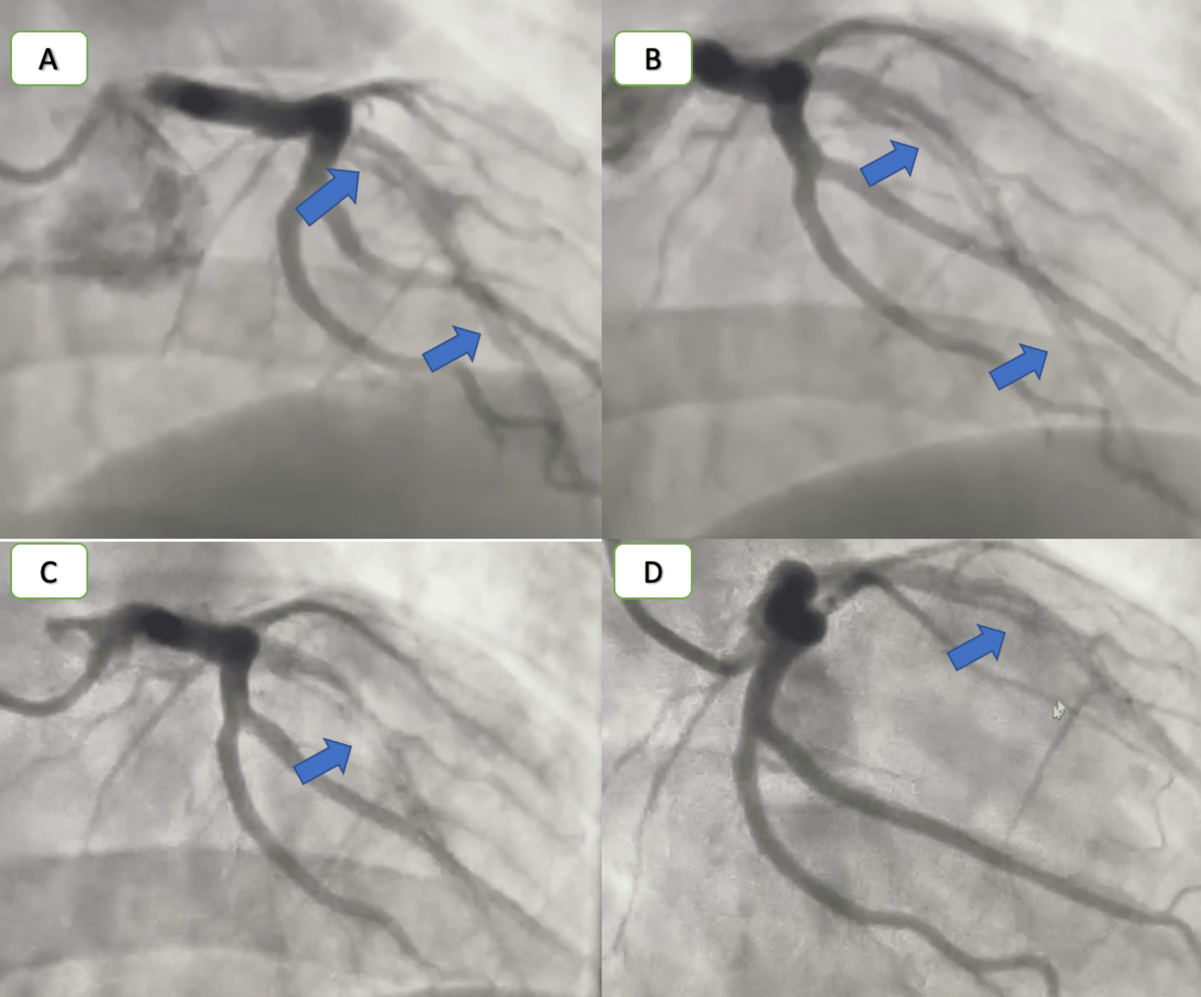
CA showing LAD dissection starting from the proximal segment and extending to the distal segment. CA, coronary angiography; LAD, left anterior descending artery

**Figure 4:**
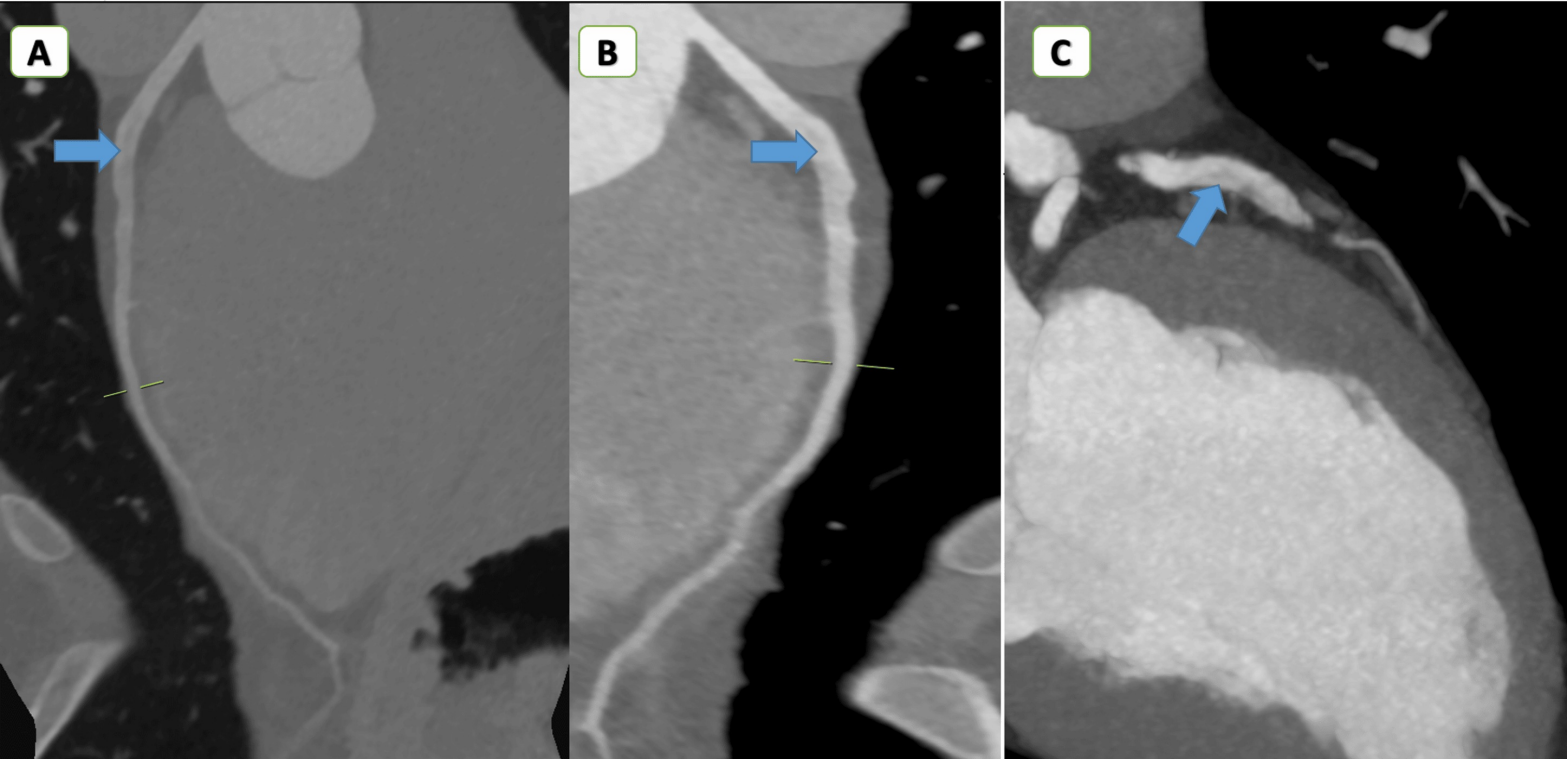
CTCA showing LAD with a dissection flap. CTCA, computed tomography coronary angiography; LAD, left anterior descending artery

Given that he had previously undergone CTCA with misdiagnosis of a normal coronary, which raised the suspicion of myocarditis, the decision was made to perform CMR to confirm the diagnosis. CMR was done on the same day, which revealed no increase in relaxation time in the T2 mapping sequence; in delayed gadolinium enhancement, there was subendocardial hyperenhancement in the apical anterior and anteroseptum (Figure 2). These findings lower the likelihood of the diagnosis of myocarditis and heighten the suspicion of coronary disease, as subendocardial enhancement is not indicative of myocarditis. We performed a CA on day two, which showed extensive dissection starting from the proximal segment and extended to the distal segment in the left anterior descending artery (LAD), which is the largest coronary artery (Figure 3). As the patient had no history of chronic medical diseases and was hemodynamically stable, the decisions involved medical treatment and follow-up. On day three, CTCA was repeated in our center to evaluate the dissection by CTCA (Figure 4).

## Discussion

Young females are more likely to have SCAD. The diagnosis of SCAD requires a high degree of suspicion, particularly in young males. Although CMR plays a limited role in the diagnosis of SCAD, it significantly altered the diagnosis in our case due to its ability to distinguish between ischemic and non-ischemic patterns in late gadolinium enhancement. While CTCA is a reliable diagnostic tool for SCAD, it requires a high level of suspicion due to its potential to miss the condition, as demonstrated in our case. Furthermore, this case highlights the significant importance of multimodality images in the diagnosis of cardiac diseases.

SCAD is an increasingly recognized cause of myocardial infarction non-obstructive coronary artery (MINOCA) by a tear in the wall of the artery not associated with risk factors either iatrogenic, traumatic, or atherosclerotic. It is rare but may lead to fatal complications (arrhythmias, ACS). The true prevalence remains unknown due to diagnostic challenges, as traditional cardiac tests are inconclusive [6].

The condition is more common in young females due to the roles of estrogen and progesterone in the connective tissue of the heart's vasculature, which increases the risk of dissection. It is also more severe and associated with more cardiac complications during pregnancy as compared to SCAD outside of pregnancy [3].

SCAD is a multifactorial disease that arises from sudden disruption of the coronary artery wall, resulting in the separation of the inner intima from the outer vessel wall. This can occur either through dissection starting in the intima, followed by a subsequent hemorrhage into the media, or through a primary hemorrhage into the media due to a rupture of the vasa vasorum, either with or without intimal injury. Oxygen deficiency occurs when damage affects the blood flow to the heart muscle through the false lumen and/or when bleeding narrows the true lumen of the coronary vessel, leading to ACS [7].

Although SCAD can affect any coronary artery, the most involved arteries are the LAD artery and its diagonal branches. The gold standard technique to confirm the diagnosis and treatment of SCAD is invasive coronary angiography, either with or without intracoronary imaging. Given that CTCA primarily allows for the assessment of proximal coronary segments and the disease primarily affects distal vessels, it is also important to consider endothelial and microvascular dysfunction [8].

Saw's classification identifies four distinct angiographic variants. Type 1 manifests as a double contour and an intimate flap. Type 2, which is the most common variant, appears as a long diffuse stenosis (>20 mm). Type 3, which is the most frequently missed because it requires intracoronary imaging, appears to be atherosclerotic stenosis. Type 4 manifests as total occlusion. Therefore, the operator's experience plays a crucial role in identifying SCAD [2].

The evidence for treatment is limited and does not rely on randomized studies. SCAD exhibits a high degree of spontaneous healing. Therefore, if the patient is symptom-free, hemodynamically stable, and the dissection location does not pose a high risk or ST elevation, goal-directed medical treatment is the most effective course of action. Stent placement or CABG is considered for patients with more proximal dissection, hemodynamic instability, dynamic ST-segment changes, and ventricular arrhythmias, Percutaneous coronary intervention (PCI) increases the risk of dissection spread and stent maladaptation during bleeding resorption. When performing PCI with stents, we administer antiplatelets as usual [2,3].

## Conclusions

While SCAD is not a common disease, it carries a risk of serious complications and is one of the significant causes of ACS that need a high degree of clinical suspicion. All imaging modalities play a significant role in diagnosing SCAD, with CA being the most commonly available invasive imaging tool. The management depends mainly on the patient's clinical and hemodynamic stability. CMR plays a crucial role in diagnosing myocardial infarction with nonobstructive coronary arteries.
